# Renal protective effects of vitamin E for drug-induced kidney injury: a meta-analysis

**DOI:** 10.3389/fphar.2025.1461792

**Published:** 2025-04-14

**Authors:** Lingfei Meng, Shengmao Liu, Wenpeng Cui

**Affiliations:** Department of Nephrology, The Second Hospital of Jilin University, Changchun, Jilin Province, China

**Keywords:** vitamin E, nephrotoxin, acute kidney injury, anti-oxidation, meta

## Abstract

**Introduction:**

Acute kidney injury (AKI) is a key clinical condition that has puzzled clinicians for many years since there is currently no efficient drug therapy. Vitamin E is found to exert a vital antioxidant role and can protect the kidney. However, clinical studies that analyze the correlation between vitamin E and AKI are scarce, and no consistent conclusions are reported from current studies. Therefore, this study was performed to evaluate the impact of vitamin E on treating AKI.

**Methods:**

The PubMed, Embase, and Cochrane Library databases were comprehensively searched on 27 December 2023. Qualified studies were selected following the eligibility criteria. The incidence of AKI, serum creatinine, and urea nitrogen levels after vitamin E treatment were evaluated. Then, the data were combined with a fixed- or random-effects model, depending on the heterogeneity test results.

**Results:**

Six eligible randomized controlled trials that used vitamin E for the prevention of kidney injury were included. According to our pooled analysis, vitamin E elevated eGFR levels [MD: 0.36; 95% CI (0.19, 0.53), p = 0.000], reduced serum creatinine levels [MD: −0.32; 95% CI (−0.48, 0.16), p = 0.000], and effectively inhibited the occurrence of AKI [RR: 0.69; 95% CI (0.49, 0.98), p = 0.036].

**Conclusion:**

Vitamin E elevates eGFR levels, reduces serum creatinine levels, and efficiently suppresses AKI occurrence.

**Systematic Review Registration:** https://www.crd.york.ac.uk/PROSPERO/view/CRD42024499597, identifier CRD42024499597

## Introduction

Acute kidney injury (AKI) is a common yet severe condition that affects millions of people and causes disability and mortality in many sufferers (Kellum et al., 2013). AKI is defined by the AKI Network by at least one or more conditions: (1) elevation of serum creatinine (Scr) level by 0.3 mg/dL (26.5 mol/L) in 48 h; (2) increase of Scr level to 1.5 folds of baseline over the past week; (3) urine volume <0.5 mL/kg/h during 6 h (Mehta et al., 2007). AKI exhibits a high and growing incidence rate globally (Hoste et al., 2018; Ronco et al., 2019). It has been reported in 7.1% of patients receiving immune checkpoint inhibitor treatment (Ji et al., 2022). In addition, 22.4% of patients develop AKI within 48 h after surgery (Gameiro et al., 2020). The presence of AKI affects acute morbidity and mortality and is related to unfavorable long-time outcomes, including an increased risk of cardiovascular complications, long-term mortality, and development of (or progression to) chronic kidney disease (CKD) (Hoste et al., 2018; Pickkers et al., 2021; James et al., 2020).

Nonetheless, effective interventions for AKI remain limited, highlighting the critical role of therapeutic drug monitoring as a cornerstone strategy in mitigating drug-associated nephrotoxicity (Ostermann et al., 2020). Based on clinical trials, early RRT implementation may not be beneficial for the survival of patients with no obvious urgent indications (Zarbock et al., 2016; Bagshaw, 2020; Gaudry et al., 2020). Implementing care bundles of AKI among inpatients can decrease the risk of moderate to severe AKI (Schaubroeck et al., 2021). AKI may be cured with early detection and treatment (Yue et al., 2023). Until now, there have been no drugs and treatments for specifically preventing or treating AKI in humans (Pickkers et al., 2021; Fang et al., 2021a).

As a fat-soluble vitamin, vitamin E exerts a critical antioxidant impact on the human body. As an essential nutrient, it was first identified by Evans and Bishop (1922) and was initially called “anti-sterility factor” or “factor X,” according to its effect on rat reproduction. It is composed of a variety of compounds, and there are eight isomers, including four tocopherols (α-, β-, γ-, and δ-tocopherol) and four tocotrienols (α-, β-, γ-, and δ-tocotrienol) in the vitamin E family (Miyazawa et al., 2019). Currently, most studies concerning vitamin E metabolism pay more attention to alpha- and gamma-tocopherols because these are bioavailable and can be more easily absorbed from diets than other vitamin E isomers (Wu and Croft, 2007).

Unstable free radical molecules may induce oxidative stress and damage cells and tissues in the body. Vitamin E, containing the chromanol hydroxyl group, can chemically scavenge free radical oxidants. Previous *in vitro* results indicate that alpha-tocopherol can prevent lipid peroxidation catalyzed by free radicals (Esterbauer et al., 1989). AKI may result from various stimuli, among which reactive oxygen species (ROS) are shown to exert a vital impact on AKI, which can induce injuries to proteins, DNA, and carbohydrates (Salehipour et al., 2010). Nonetheless, vitamin E may mitigate AKI symptoms (Koga et al., 2012; Kataoka et al., 2012; Liu et al., 2015). Additionally, emerging mechanistic studies indicate that vitamin E significantly suppresses ROS and ameliorates AKI (Zhang et al., 2024; Georgiev et al., 2023). Apart from its antioxidant effect, vitamin E can regulate gene expression, resist inflammation, and protect the kidneys (Zaaboul and Liu, 2022).

The conclusions drawn from current studies on the role of vitamin E in protecting AKI are still inconsistent (Cho et al., 2017; Xu et al., 2018; Monami et al., 2021). To our knowledge, there is currently no meta-analysis that explores the effect of vitamin E on drug-induced AKI. Therefore, this study aimed to investigate the correlation between vitamin E and drug-induced AKI in randomized controlled trials (RCTs) performed with humans.

## Methods

### Search methods

The PubMed, Cochrane Library, and Embase databases were searched from inception to 27 December 2023 to find published RCTs on the use of vitamin E in high-risk AKI patients with available kidney function outcomes. Keywords included “contrast induced kidney injury,” “renal ischemia reperfusion injury,” “acute renal failure,” “nephrotoxicity,” “AKI,” “kidney damage,” “renal function,” “vitamin E,” “tocopherols,” “tocopherylquinone,” “tocotrienols,” and “tocophersolan.” The research was limited to human studies, and only English language manuscripts were included. Abstracts of all articles were reviewed to develop a full reference list. In addition, references in related original and review studies were checked to manually identify qualified RCTs. This study is registered with PROSPERO (number CRD42024499597).

### Study selection

In the current meta-analysis, two investigators (Lingfei Meng and Shengmao Liu) independently conducted title- and abstract-screening of eligible publications. To identify whether these studies satisfied our preset eligibility criteria, the full texts of those screened articles were downloaded. Any disagreements between them were settled by mutual negotiation with a third investigator (Wenpeng Cui).

### Eligibility criteria

#### Inclusion criteria

The inclusion criteria were (1) RCTs with a crossover or parallel design; (2) studies exploring the role of vitamin E in kidney function in human; (3) studies including patients aged ≥14 years; (4) the use of vitamin E in the treatment group; (5) studies setting a placebo or control group; (6) studies with primary outcomes of Scr, blood urea nitrogen (BUN), eGFR, or AKI; (7) studies published in English.

#### Types of studies

RCTs evaluating the application of vitamin E in renoprotection in humans were included.

#### Types of study populations

The study populations included adults with AKI associated with drug application in humans.

#### Types of interventions

All vitamin E types compared with control or placebo were involved, with no limitation of dosage, dosage form, vitamin E application time, or duration.

#### Types of outcomes

Primary outcomes were probabilities of AKI, eGFR, Scr, and BUN to evaluate kidney function.

#### Exclusion criteria

The following exclusion criteria were applied: (1) studies in which vitamin E was accompanied by other interventions, uncontrolled clinical trials, conference abstracts, book chapters, comments, interviews, opinion pieces, position papers, methodological papers, letters, editorials, and animal or cell culture studies; (2) studies irrelevant to drugs related kidney injury; (3) studies not reporting relevant outcomes, mean or median and standard deviation (SD), and the number of AKI episodes; (3) studies with *in vitro* experiments and animal experiments alone; and (4) non-available articles.

#### Data collection

Data were collected from eligible RCTs by two investigators (Lingfei Meng and Shengmao Liu) independently. Any disagreements between them were settled by negotiation with a third investigator (Wenpeng Cui). These relevant data were collected from each study (Table 1): (1) study features (first author, publication year); (2) study population; (3) interventions (grouping, dosage and time, route, timing, and duration of administration); and (4) eGFR, BUN, and Scr data, as well as the number of AKI episodes. In addition, the means, SD, AKI episodes, and population size in each group were collected.

**TABLE 1:** Characteristics of primary analysis of the included trials.

Year	Author	Study design	Age	Duration	Study population	Group	Intervention	Sample size	AKI	SCR(mean ± SD), μmol/L	BUN(mean ± SD), mmol/L	eGFR, mL/min
2020	Farzaneh Ashrafi	RCT	Age ≥18 years	3 weeks	Cisplatin-based chemotherapy candidates	Treatment	400 IU vitamin E daily	26		66.3 ± 8.84		107.6 ± 18
						Control	Placebo	25		75.14 ± 17.68		93.3 ± 24.6
2023	Mahdi Moradi Goudarzi	RCT	Aged 14–60	30 days	Patients with thalassemia major	Treatment	400 IU vitamin E daily	23				162.77 ± 67.6
						Control	No treatment	27				132.58 ± 35.45
2021	Maryam Samsami	RCT	Age ≥18 years old	At least 7 days	Patients underwent colistin therapy	Treatment	400 IU alpha-tocopherol daily	26	12			
						Control	No treatment	26	11			
2013	Adis Tasanarong	RCT	Adult	48 h	CKD patients with contrast media	Treatment	α-Tocopherol (350 mg/day)	102	13	140.56 ± 53.92		47 ± 16
						Treatment	γ-Tocopherol (300 mg/day)	102	16	133.48 ± 92.11		49 ± 19
						Control	Placebo	101	24	1.77 mg/dL ± 0.85		43 ± 17
2012	Thomas M. Kitzler	RCT	Age ≥18 years old	12 h and 6 h before and after the CT	CKD with contrast-induced kidney injury	Treatment control	2060 mg vitamin E emulsion	9		120.22 ± 19.45		50.9 ± 11.3
							Placebo emulsion	10		120.22 ± 9.72		45.6 ± 5.8
2009	Adis Tasanarong	RCT	Adult	48, 24 h, and the morning before coronary procedures	The patients with contrast media	Treatment	525 IU alpha-tocopherol daily	51		144.98 ± 52.16	22 ± 9	
						Control	Placebo	52		167.96 ± 76.91	27 ± 13	

#### Assessment of risk of bias

To examine the overall study quality, two reviewers (Lingfei Meng and Shengmao Liu) assessed the bias risk using RevMan 5.3. Bias was marked as high, low, or “unclear” (indicating that the bias risk was unknown). Any disagreement was settled by discussion with a third reviewer (Wenpeng Cui).

#### Statistical analysis

If two or more studies presented the findings of an identical outcome, we summarized data and assessed the outcomes (eGFR, BUN, Scr, and AKI). Initially, we performed meta-analysis of articles comparing vitamin E with control groups, using Review Manager 5.3 software and Stata SE 15 for data analysis. The results presented in the current report were mean difference (MD) of measurements with the same unit or standardized mean difference (SMD) with different units. To evaluate the therapeutic effect of vitamin E, the pooled data were employed. In addition, I^2^ statistic was used to assess heterogeneity among our enrolled articles. A fixed-effect model should be applied in the case of insignificant heterogeneity (i.e., p > 0.1; I^2^ ≤ 50%); otherwise, a random-effect model was applied.

## Results

### Characteristics of eligible studies

**FIGURE 1:**
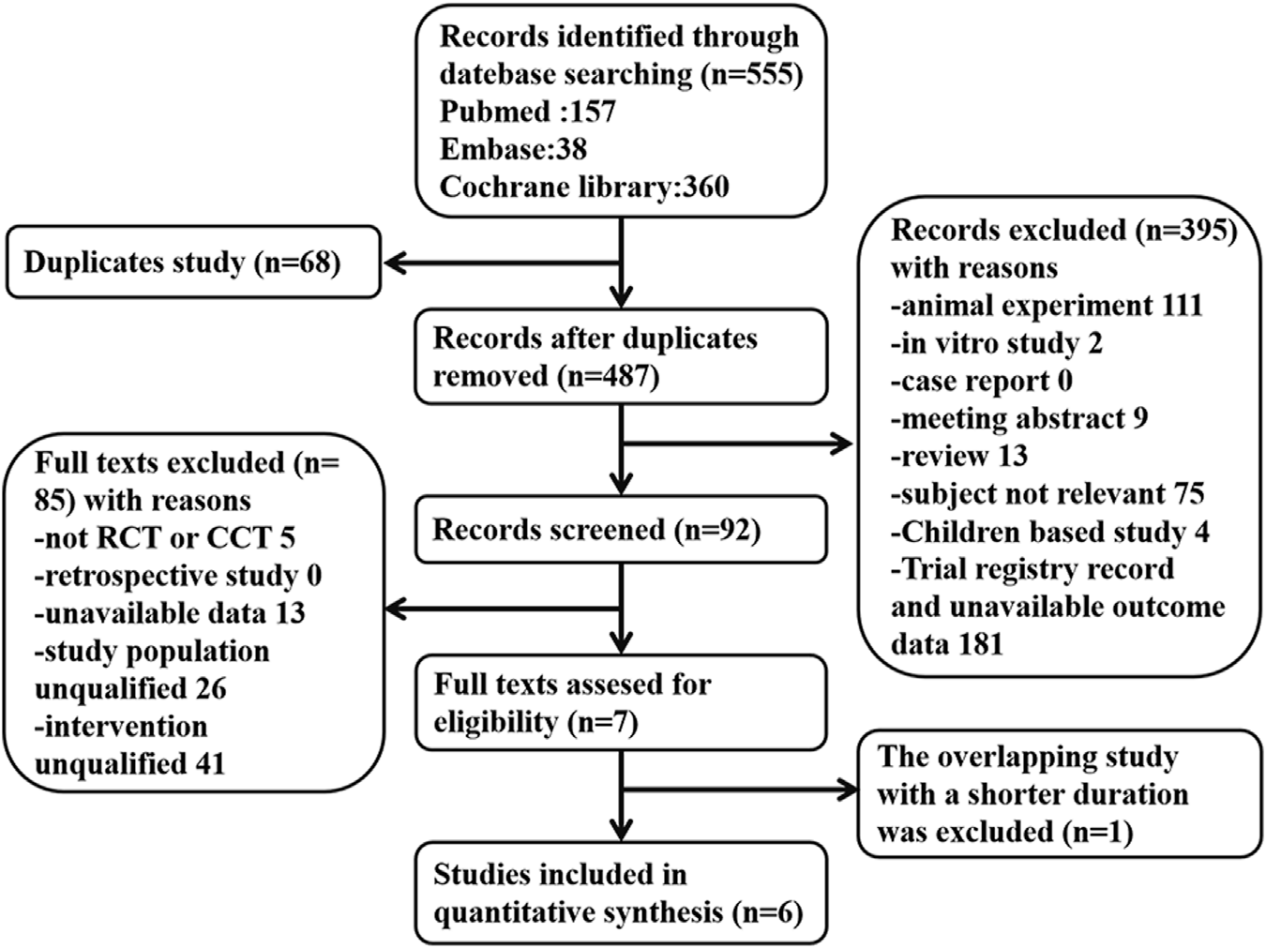
Flow diagram and description of the study selection process.

A total of 555 articles were acquired through literature search and later screened following the inclusion and exclusion criteria. If two reports covered overlapping studies, the one involving more samples and/or having a longer study period was chosen. There were six eligible RCTs concerning vitamin E for the prevention of kidney injury. Of these trials, kidney function was applied as the primary or secondary outcome. Contrast media-induced kidney injury was reported in three RCTs. Other drug-induced kidney injuries were reported in three RCTs. AKI was reported in two studies, while eGFR, Scr, and BUN were reported in four, four, and one study, respectively. Figure 1 summarizes the screening process of eligible clinical trials. Finally, six RCTs involving 580 participants were included for analysis. All patients in these studies received vitamin E treatment, and the follow-up time in all studies ranged from 1 day to 2 months. Table 1 presents the characteristics of the studies we included on analyzing kidney protection.

### Quality assessment

**FIGURE 2:**
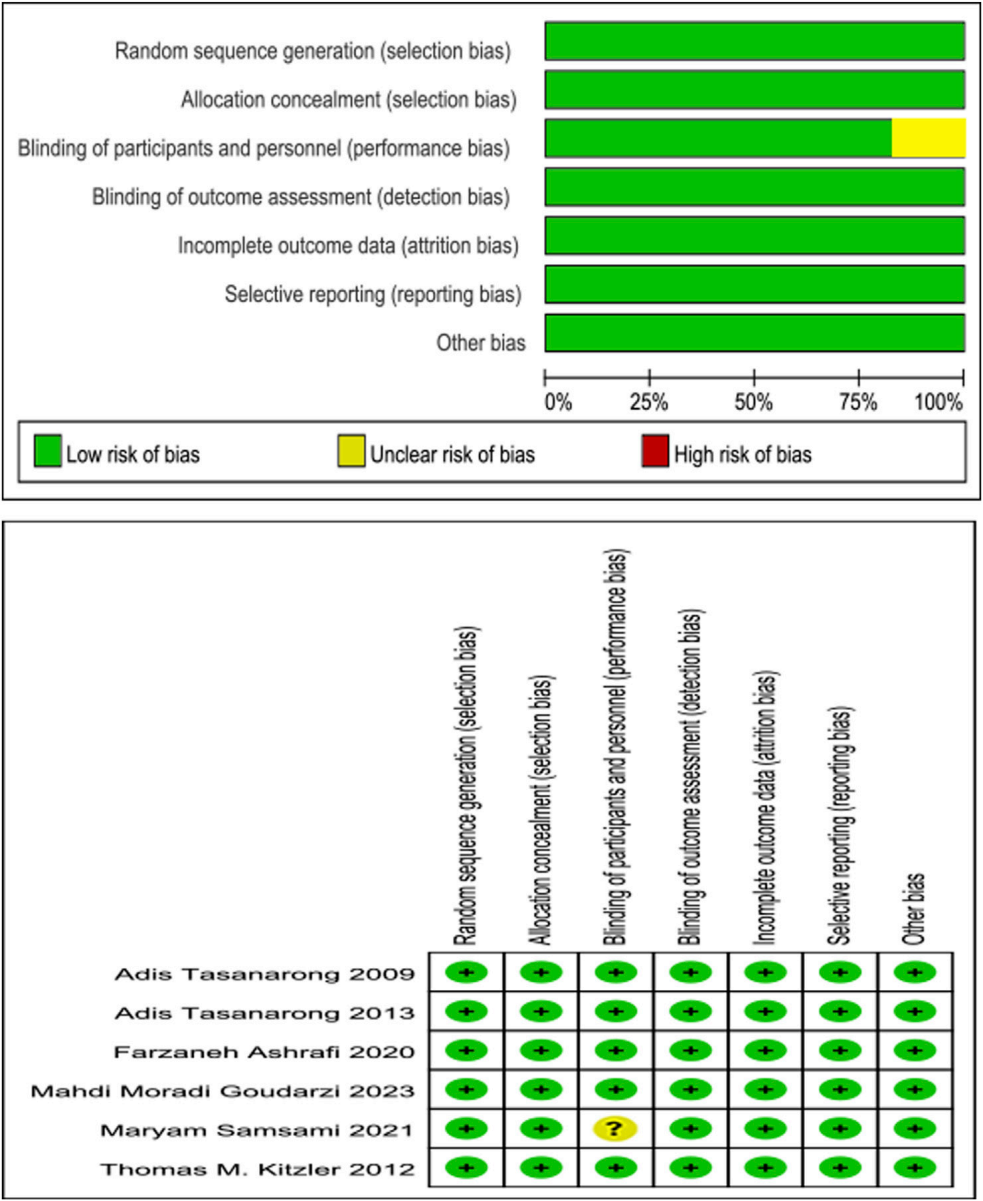
Chart and summary of the bias risk.

The methodological features most associated with the risk of bias below were assessed, including randomization, allocation concealment, treatment allocation blinding, outcome assessment blinding, incomplete outcome data and selective reporting, and other biases. With study quality, five of these six enrolled RCTs met seven of the above criteria, and one of these six enrolled RCTs met six (Figure 2).

### Effects of interventions

#### Primary outcome: AKI

**FIGURE 3:**
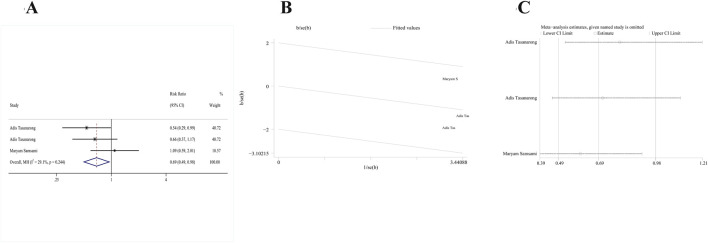
Comparison of efficacy between vitamin E and placebo in AKI. **(A)** Forest map. **(B)** Test of heterogeneity by the galbr plot. **(C)** Sensitivity analysis.

Two RCTs (Tasanarong et al., 2013; Samsami et al., 2021) satisfied our inclusion criteria. One study included both α- and γ-tocopherol in the treatment group, and thus study data were extracted twice. Due to the absence of significant heterogeneity, a fixed-effects model (p = 0.244, I^2^ = 29.1%) was used. After combined analysis of these two studies, the AKI incidence of the vitamin E group significantly decreased compared to the control group [RR: 0.69; 95% CI (0.49, 0.98), p = 0.036] (Figure 3A). The galbr plot exhibited little heterogeneity between the three outcomes of the two studies (Figure 3B). Based on sensitivity analysis, no study exerted a disproportionate impact on the outcomes (Figure 3C).

#### Primary outcome: eGFR

**FIGURE 4:**
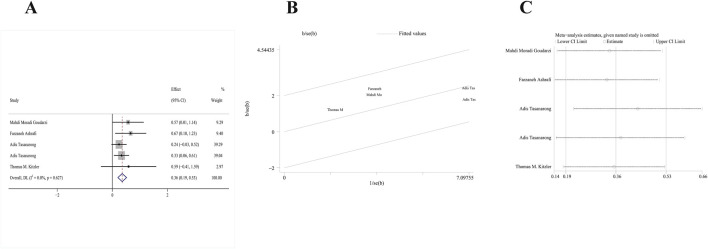
Comparison of efficacy between vitamin E and placebo in eGFR. **(A)** Forest map. **(B)** Test of heterogeneity by the galbr plot. **(C)** Sensitivity analysis.

Four RCTs (Tasanarong et al., 2013; Goudarzi et al., 2023; Ashrafi et al., 2020; Kitzler et al., 2012) met our inclusion criteria. One study included both α- and γ-tocopherol in the treatment group; thus, study data were extracted twice. A fixed-effects model was employed since no significant heterogeneity was detected (p = 0.627, I^2^ = 0%). According to pooled results from these four RCTs, the change of serum eGFR from baseline to follow-up of vitamin E group significantly decreased compared with the control group [MD: 0.36; 95% CI (0.19, 0.53), p = 0.000] (Figure 4A). In addition, the galbr plot showed little heterogeneity between the included studies (Figure 4B). According to sensitivity analysis, no study exerted a disproportionate effect on the outcome (Figure 4C).

#### Primary outcome: Scr

**FIGURE 5:**
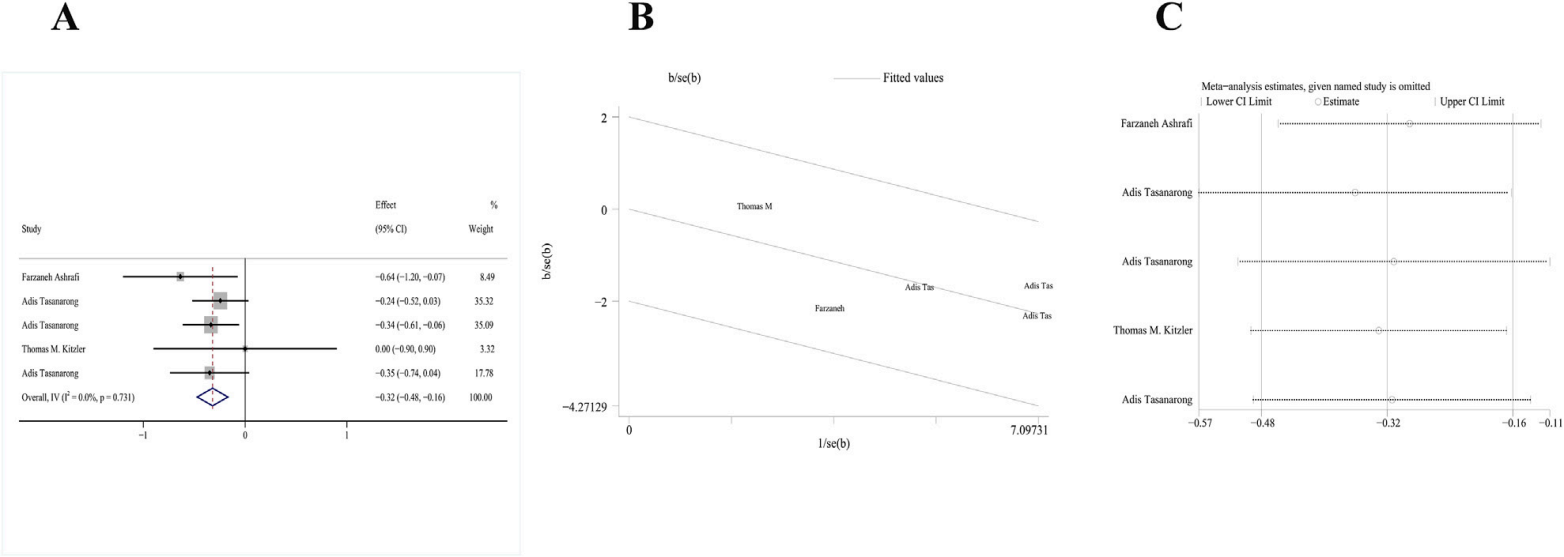
Comparison of efficacy between vitamin E and placebo in Scr. **(A)** Forest map. **(B)** Test of heterogeneity by the galbr plot. **(C)** Sensitivity analysis.

Four RCTs (Tasanarong et al., 2013; Ashrafi et al., 2020; Kitzler et al., 2012; Tasanarong et al., 2009) mentioning the role of vitamin E in Scr as the primary outcome satisfied our inclusion criteria. The serum creatinine follow-up data were extracted. A fix-effects model was used owing to the absence of heterogeneity (p = 0.731, I^2^ = 0.00%). The pooled analysis of these studies demonstrated that the change of serum creatinine level in the treatment group between baseline and follow-up significantly decreased relative to the control group [MD: −0.32; 95% CI (−0.48, 0.16), p = 0.000] (Figure 5A). The galbr plot exhibited little heterogeneity between the included studies (Figure 5B). Sensitivity analysis indicated that the serum creatinine data in the included RCTs remained stable (Figure 5C).

#### Primary outcome: BUN

One RCT (Tasanarong et al., 2009) mentioned the role of vitamin E in serum BUN as the primary outcome. No data extraction or analysis was performed.

## Discussion

This meta-analysis enrolled six RCTs about vitamin E for AKI. The designs of these studies were reasonable, and they were of high quality. Our pooled analysis indicated that vitamin E effectively inhibited the occurrence of AKI, increased the eGFR level, and reduced the Scr level in drug-induced kidney injury.

AKI has puzzled clinicians as its pathophysiology has not been completely understood. There are multiple mechanisms involved in its process, including oxidative stress, inflammation, RAAS activation, and DNA damage (De Chiara et al., 2022; Schefold et al., 2016). Among these, oxidative stress exerts a vital role in AKI. According to related basic and clinical study results, vitamin E makes an anti-oxidative stress impact on AKI. It also shows a protective effect against nephrotoxicity induced by drugs like cisplatin or other nephrotoxic medicines.

Vitamin E prevents drug-mediated kidney damage. Numerous animal experiments regarding the therapeutic efficacy of vitamin E in cisplatin-related toxicities have been published (Abdel-Daim et al., 2019; Abo-Elmaaty et al., 2020; Busari et al., 2018). Cisplatin-related toxicities may mostly result from free radical generation, and the latter can induce oxidative organ injury (Hakiminia et al., 2019). ROS production in mitochondria is a major mechanism related to nephrotoxicity. ROS production and proapoptotic protein activation within an intrinsic pathway can lead to increased mitochondrial membrane permeability (Servais et al., 2008) which reduces the mitochondrial membrane potential, causes calcium homeostasis imbalance, decreases adenosine triphosphate (ATP) generation, and leads to mitochondrial respiratory chain impairment (dos Santos et al., 2012). Vitamin E can increase ATP levels in every tissue except muscle and improve drug-induced mitochondrial dysfunctions in rats (Eser Faki et al., 2020).

Vitamin E also has a protective impact on kidney damage induced by other drugs. Its use can improve oxidative stress indicators, inflammatory markers, histopathological results, and kidney functions in rats with toxic acetamiprid (Erdemli et al., 2020). Vitamin E supplementation can mitigate cadmium-mediated oxidative stress and injury in rat kidneys by suppressing renal cell apoptosis and promoting the antioxidant defense system (Fang et al., 2021b). Supplementation with vitamins E alone in rats applied with amphotericin B can improve kidney tissue structures, functional parameters, and oxidative stress status (Salehzadeh et al., 2020). Vitamin E can regulate apoptosis and autophagy to prevent HgCl_2_-mediated kidney injury (Alhusaini et al., 2022). Furthermore, vitamin E can be used to mitigate kidney impairments induced by chronic gabapentin use, and its possible mechanism is associated with the inhibition of tissue injury and apoptosis biomarkers (Welson et al., 2021).

The use of vitamin E in treating AKI thus has a profound pathophysiological basis. Human studies on the application of vitamin E in treating AKI are limited, and no consistent conclusion has been drawn (Samsami et al., 2021; Goudarzi et al., 2023; Kitzler et al., 2012; Zhao et al., 2018; Koay et al., 2021). Although some systematic reviews on contrast-induced AKI (CIAKI) have been performed, the conclusions are also inconsistent (Cho et al., 2017; Xu et al., 2018; Monami et al., 2021; Cho et al., 2017). One meta-analysis showed that vitamin E significantly decreased the CIAKI risk ratio by 62% and reduced Scr elevation following contrast application; however, no differences were found in GFR (Cho et al., 2017). Another meta-analysis showed that α-tocopherol pretreatment prior to contrast medium-requiring imaging examinations significantly reduced contrast-induced nephropathy risk, while it exerted no impact on Scr or eGFR (Monami et al., 2021). The meta-analysis performed by Xu et al. (2018) found that vitamin E was beneficial for decreasing CIAKI risk. These studies’ conclusions are probably inconsistent due to their heterogeneous timing and inclusion criteria and their outcome definitions. Our meta-analysis offers some strengths as the sample size increases as the study population focuses on patients with drug-induced AKI.

Four studies regarding the impact of vitamin E on Scr were included in our meta-analysis (Kitzler et al., 2012; Tasanarong et al., 2009; Schefold et al., 2016). In other clinical trials, one study showed that vitamin E, compared to placebo, significantly decreased Scr levels in patients with diabetic nephropathy (Tan et al., 2018), supporting our conclusion. Another study found that vitamin E and allopurinol did not significantly decrease Scr levels among patients developing pre-existing kidney failure who received coronary artery bypass grafting (CABG) surgery (Nouri-Majalan et al., 2009). This seemed inconsistent with our conclusion. The inconsistency in the findings may be related to the inclusion of interventions other than vitamin E alone and difference in the study population. Our meta-analysis only included vitamin E not accompanied by other treatments, which is more beneficial.

As was concluded in four RCTs about the function of vitamin E in eGFR, vitamin E effectively increased eGFR levels. Patients with drug-induced AKI were studied in this meta-analysis. However, most clinical trials have focused on vitamin E and eGFR in diabetic kidney disease. One RCT showed that compared with placebo, vitamin E did not influence eGFR in patients with diabetic nephropathy at 2 months (Tan et al., 2018). Another study indicated that vitamin E application led to improved kidney function at 8 and 12 months, as evaluated through eGFR in diabetic kidney disease (Koay et al., 2021). The course of vitamin E was different in the above two studies. A longer-term application of vitamin E had better renoprotection. Nevertheless, vitamin E may have an inconsistent function in people with different genotypes of diabetes. Based on Dalan et al. (2020), vitamin E application resulted in increased eGFR levels in patients with haptoglobin 2-2 (Hp2-2) genotype diabetes compared with the non-Hp2-2 group. A newly published meta-analysis reported that eGFR of the vitamin E group did not exhibit any statistical significance in diabetic nephropathy (Jin et al., 2024). Furthermore, the pathogenesis of diabetic nephropathy involves multiple interconnected pathways, such as hemodynamic disturbances, oxidative stress, cellular apoptosis, and genetic/epigenetic regulation, with glomerular lesions representing the most prominent pathological feature (Wang et al., 2023). In contrast, drug-induced kidney injury, although it also involves oxidative stress and other mechanisms, primarily manifests as tubulointerstitial damage, a leading contributor to AKI (Perazella and Rosner, 2022). These distinct pathogenic mechanisms, combined with the heterogeneity of study populations, may explain the differential effects of vitamin E on eGFR.

In two RCTs included in this study, the vitamin E group showed a significantly reduced AKI risk, although no significant difference was found in AKI prevalence and duration between vitamin E and control groups in Samsami et al. (2021). Currently, vitamin E combined with additional drugs attracts more attention. Guo and Wang (2018) suggested that vitamin E combined with umbilical cord mesenchymal stem cells (UC-MSC) apparently suppressed the renal inflammatory response by regulating inflammatory cytokines within the kidney microenvironment in AKI rats. They also found that vitamin E combined with UC-MSC has superior efficacy in treating AKI to vitamin E or UC-MSC monotherapy. Truksa et al. (2015) reported that vitamin E analogs targeting mitochondria represent a new mitocan class, which can cause apoptosis and inhibit proliferation, transcription and normal mitochondrial activity. Regulating mitochondrial activity with the mitochondria-targeting antioxidant is the candidate way to mitigate injury of proximal tubular epithelial cells (Sakamoto et al., 2017). Site-targeting vitamin E is also a vital direction in the future of AKI. Due to the limited number of studies included, caution is warranted when generalizing the findings regarding the therapeutic effect of vitamin E on AKI. Further large-scale studies are needed to validate our conclusions.

Nevertheless, as reported in some clinical studies, high-dose α-tocopherol supplementation induces unfavorable effects (Brown et al., 2001; Ward et al., 2007), which is also supported by a meta-analysis (Miller et al., 2005). The dosage of vitamin E therapy is an important issue. Clearly, the mean dietary source of α-tocopherol is approximately 1–10 mg^68^. Oral vitamin E can be absorbed by the body and catabolized in the liver and intestine. In an intervention with 40% fat, it can be estimated that most metabolite excreted in urine is derived from the liver. In the fasting period, nearly half of metabolite is derived from the liver, while the rest is from the intestine (Traber et al., 2021). Nevertheless, research has not yet identified the clinically effective blood concentration of biologically active vitamin E for ameliorating AKI. Concerning serum levels, 13% of all recruited data points worldwide are below 12 μmol/L, which is the threshold of functional deficiency and is mainly for neonates and children. Based on some prospective observational studies, the α-tocopherol content ≥30 μmol/L in serum benefits human health (Péter et al., 2015). The excessive use of vitamin supplements containing high-dose vitamins A, D, and E may lead to AKI (De Francesco Daher et al., 2017). Both the dose of vitamin E therapy and target serum concentration will be directions of future studies.

The strength of our study is that all the included studies were high-quality RCTs with a relatively large population. As far as we know, this is the first meta-analysis exploring the effect of vitamin E on drug-induced AKI in RCTs performed in humans. Nonetheless, this study does have the following limitations: differences in the agents causing AKI, the inclusion of patients with varying underlying conditions, variations in dosing regimens, interventions in the control groups, and follow-up periods that may have contributed to heterogeneity. Due to the limiting data from the included RCTs, the side effects of vitamin E were not analyzed systematically. Therefore, more RCTs are needed to study the therapeutic effects of different vitamin E doses, the effect of vitamin E on markers of oxidative stress, and the side effects of vitamin E.

## Conclusion

Vitamin E can effectively inhibit the occurrence of AKI, increase eGFR levels, and decrease Scr levels. Further well-designed RCTs should be performed to verify the protection, side effects, and appropriate dosing of vitamin E.

## Data Availability

The original contributions presented in the study are included in the article/Supplementary Material; further inquiries can be directed to the corresponding author.
